# Metabolic engineering of *Escherichia coli* for production of mixed isoprenoid alcohols and their derivatives

**DOI:** 10.1186/s13068-018-1210-0

**Published:** 2018-07-24

**Authors:** Bakht Zada, Chonglong Wang, Ji-Bin Park, Seong-Hee Jeong, Ju-Eon Park, Hawaibam Birla Singh, Seon-Won Kim

**Affiliations:** 1 Division of Applied Life Science (BK21 Plus Program), PMBBRC, Gyeongsang National University, Jinju, 52828 Republic of Korea; 2 School of Biology and Basic Medical Sciences, Soochow University, Suzhou, People’s Republic of China

**Keywords:** Isoprenoid mixture, Isopentenol, Geraniol, Farnesol, *Escherichia coli*, NudB

## Abstract

**Background:**

Current petroleum-derived fuels such as gasoline (C_5_–C_12_) and diesel (C_15_–C_22_) are complex mixtures of hydrocarbons with different chain lengths and chemical structures. Isoprenoids are hydrocarbon-based compounds with different carbon chain lengths and diverse chemical structures, similar to petroleum. Thus, isoprenoid alcohols such as isopentenol (C_5_), geraniol (C_10_), and farnesol (C_15_) have been considered to be ideal biofuel candidates. NudB, a native phosphatase of *Escherichia coli*, is reported to dephosphorylate isopentenyl diphosphate (IPP) and dimethylallyl diphosphate (DMAPP) into isopentenol. However, no attention has been paid to its promiscuous activity toward longer chain length (C_10_–C_15_) prenyl diphosphates.

**Results:**

In this study, the promiscuous activity of NudB toward geranyl diphosphate (GPP) and farnesyl diphosphate (FPP) was applied for the production of isoprenoid alcohol mixtures, including isopentenol, geraniol, and farnesol, and their derivatives. *E. coli* was engineered to produce a mixture of C_5_ and C_15_ alcohols by overexpressing NudB (dihydroneopterin triphosphate diphosphohydrolase) and IspA (FPP synthase) along with a heterologous MVA pathway, which resulted in a total of up to 1652 mg/L mixture of C_5_ and C_15_ alcohols and their derivatives. The production was further increased to 2027 mg/L by overexpression of another endogenous phosphatase, AphA, in addition to NudB. Production of DMAPP- and FPP-derived alcohols and their derivatives was significantly increased with an increase in the gene dosage of *idi*, encoding IPP isomerase (IDI), indicating a potential modulation of the composition of the alcohols mixture according to the expression level of IDI. When IspA was replaced with its mutant IspA*, generating GPP in the production strain, a total of 1418 mg/L of the isoprenoid mixture was obtained containing C_10_ alcohols as a main component.

**Conclusions:**

The promiscuous activity of NudB was newly identified and successfully used for production of isoprenoid-based alcohol mixtures, which are suitable as next-generation biofuels or commodity chemicals. This is the first successful report on high-titer production of an isoprenoid alcohol-based mixture. The engineering approaches can provide a valuable platform for production of other isoprenoid mixtures via a proportional modulation of IPP, DMAPP, GPP, and FPP syntheses.

**Electronic supplementary material:**

The online version of this article (10.1186/s13068-018-1210-0) contains supplementary material, which is available to authorized users.

## Background

Petroleum-derived fuels used for transportation are complex mixtures of carbon compounds with different chain lengths and chemical structures. For example, gasoline, diesel, and jet fuels are mixture of hydrocarbons with carbon number distribution from 5 to 12, 15–22, and 8–16, respectively. With the increasing concern about the limited supply of petroleum and the effects of climate change [1], interest in researching microbial production of sustainable and renewable biofuels has increased [2]. Advanced biofuels are required to have similar fuel properties to petroleum-derived gasoline, diesel, or jet fuels in order to replace the current fuels [3–5].

Isoprenoids, also known as terpenoids, are a large and diverse class of natural compounds used as pharmaceuticals, fragrances and flavors, solvents, and more recently, potential advanced biofuels [6–9]. Isopentenol (C_5_H_10_O, also known as isoprenol and prenol), geraniol (C_10_H_18_O), and farnesol (C_15_H_26_O) are important acyclic hemiterpene, monoterpene, and sesquiterpene alcohols, respectively. These alcohols are considered to be advanced biofuels due to their high-energy content, low hygroscopicity, and low volatility [2, 10–13]. Additionally, they have a wide range of commercial applications as flavor, fragrance, pharmaceuticals, biopesticides, and precursors to important industrial chemicals [7, 14–23]. Isopentenol, geraniol, and farnesol are biologically generated from prenyl diphosphates precursors, IPP/DMAPP, GPP, and FPP, respectively. IPP and DMAPP are synthesized via the methylerythritol phosphate (MEP) pathway, starting from condensation of pyruvate and glyceraldehyde-3-phosphate (G-3-P) in most prokaryotes including *E. coli*, and the mevalonate (MVA) pathway, commencing with condensation of three acetyl-CoA in eukaryotes [24, 25]. GPP and FPP are synthesized by an isoprenyl diphosphate synthase such as IspA via a sequential head-to-tail condensation of IPP and DMAPP.

The dephosphorylation of IPP/DMAPP, GPP, and FPP to isopentenol, geraniol, and farnesol, respectively can be catalyzed by various phosphatases, diphosphatases, and terpene synthases. Some of *E. coli* endogenous phosphatases efficiently catalyze the dephosphorylation of IPP and DMAPP to isopentenol [13], but an effective native enzyme, catalyzing GPP and FPP to geraniol and farnesol, respectively, has not yet been identified. Exogenous geraniol synthase from *Ocimum basilicum* has been expressed for geraniol production in *E. coli* [26–28]. Bifunctional diacylglycerol diphosphate phosphatase (DPP1) and lipid phosphate phosphatase (LPP1) from *Saccharomyces cerevisiae* have been evaluated for geraniol biosynthesis [29]. The endogenous *E. coli* ADP-ribose pyrophosphatase (NudF) and alkaline phosphatase (PhoA) were also tested for geraniol production; however, only a tiny amount (5.3 mg/L) of geraniol was produced by PhoA in a flask culture [29]. Farnesyl phosphatases from *Homo sapiens* and *Drosophila melanogaster* can hydrolyze FPP to farnesol [30, 31]. Farnesol synthase from *Oryza sativa* and sesquiterpene synthase from *Zea mays* catalyze the conversion of FPP to farnesol [32, 33]. Several phosphatases from *S. cerevisiae* such as a truncated alkaline phosphatase, diacylglycerol diphosphate phosphatase, and lipid phosphate phosphatase show specificity toward FPP [34, 35]. *E. coli* native phosphatases, PhoA, AphA, BacA, PgpA, and PgpB, have also been assessed for farnesol synthesis [36, 37]. However, the enzymes investigated for farnesol synthesis have low substrate specificity, low catalytic activity, or poor protein expression in microbial hosts. Thus, the discovery of a promising candidate enzyme that can effectively catalyze dephosphorylations of prenyl diphosphates to their corresponding alcohols is still needed.

**Fig. 1:**
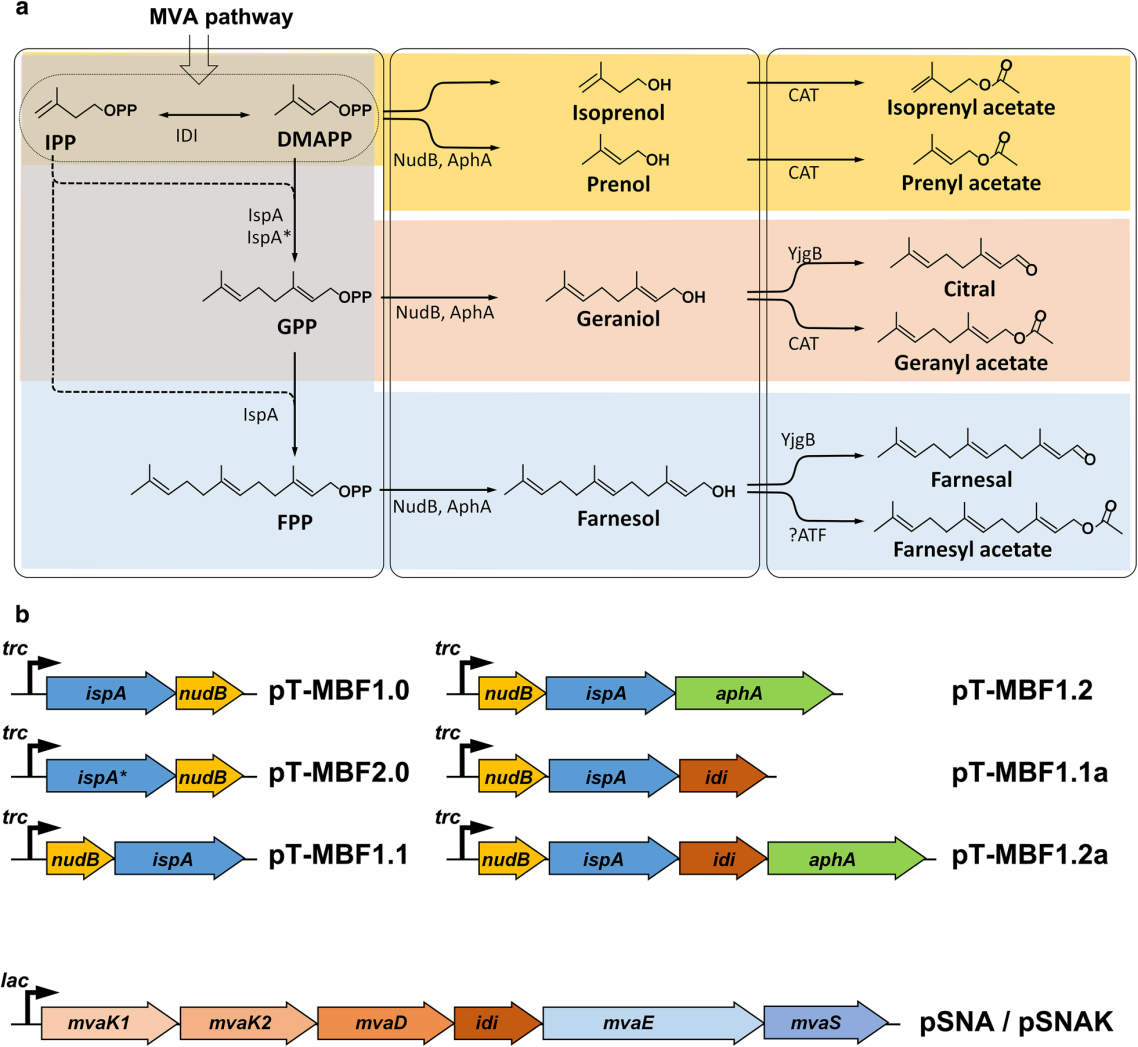
Schematic overview of synthesis pathway (**a**) and expression constructs containing the pathway genes (**b**). GPP and FPP are synthesized by IspA* and IspA, respectively, through condensations of DMAPP and IPP, generated from the MVA pathway. NudB catalyzes dephosphorylation of IPP, DMAPP, GPP, and FPP into IP, DMAP, GP, and FP, respectively. AphA catalyzes hydrolysis of IP, DMAP, GP, and FP into isoprenol, prenol, geraniol, and farnesol, respectively. Chloramphenicol acetyltransferase (CAT) is responsible for transformation of isoprenol, prenol, and geraniol into their corresponding acetate esters. An alcohol dehydrogenase, YjgB oxidizes geraniol and farnesol into citral and farnesal, respectively. An unknown acetyl transferase (ATF) is involved in transformation of farnesol into farnesyl acetate. Solid colored arrows and black bent arrows represent genes and promoters, respectively. Abbreviations of the pathway intermediates are as follows: MVA, mevalonate; IPP, isopentenyl diphosphate; DMAPP, dimethylallyl diphosphate; GPP, geranyl diphosphate; FPP, farnesyl diphosphate; IP, isopentenyl phosphate; DMAP, dimethylallyl phosphate; GP, geranyl phosphate; FP, farnesyl phosphate. The abbreviations of enzymes are as follows: NudB, dihydroneopterin triphosphate diphosphatase; IspA, farnesyl diphosphate synthase; AphA, acid phosphatase; IspA*, IspA mutant of geranyl diphosphate synthase; MvaE, bifunctional acetoacetyl-CoA thiolase and HMG-CoA reductase; MvaS, HMG-CoA synthase; MvaK1, mevalonate kinase; MvaK2, phosphomevalonate kinase; MvaD, mevalonate diphosphate decarboxylase; IDI, isopentenyl diphosphate isomerase; CAT, chloramphenicol acetyltransferase; YjgB NADPH-dependent aldehyde reductases; ?ATF, unknown acetyl transferase of *E. coli*

Nudix hydrolases, which are distributed among all kingdoms of life, potentially recognize and dephosphorylate phosphate moieties of a wide range of phosphorylated compounds [38, 39]. Nudix hydrolases from *Bacillus subtilis* (BsNudF) and *E. coli* (NudB, NudF, NudI, NudJ, and NudM) convert IPP and DMAPP into isopentenol [13, 40]. As Nudix hydrolases have been suspected to dephosphorylate isopentenyl diphosphates to isoprenoid alcohols, the highly active NudB is used here for production of a mixture of isoprenoid alcohols including geraniol, farnesol, and isopentenol (Fig. 1a).

## Results and discussion

### Engineering a synthetic pathway for a mixture of isoprenoid-based C_5_ and C_15_ alcohols

**Fig. 2:**
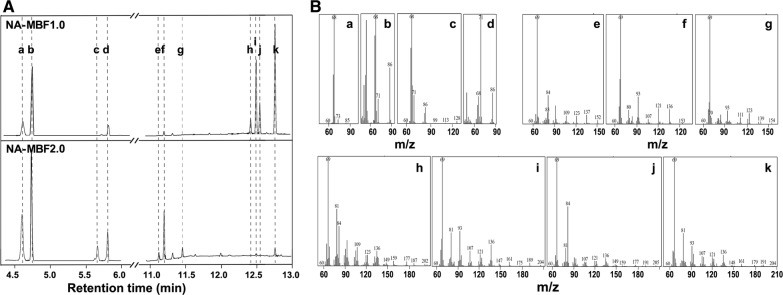
GC-FID and GC–MS analysis of C_5_, C_10_, and C_15_ isoprenoid alcohols and their derivatives from engineered *E. coli* strains. GC-FID chromatograms (**A**) and mass spectra (**B**) of culture extracts obtained from recombinant *E. coli* strains NA-MBF1.0 and NA-MBF2.0. The GC-FID peaks and their corresponding compound are a isoprenyl acetate; b isoprenol; c prenyl acetate; d prenol; e citral; f geranyl acetate; g geraniol; h *Z*,*E*-farnesal; i farnesyl acetate; j *E*,*E*-farnesal; and k farnesol

**Fig. 3:**
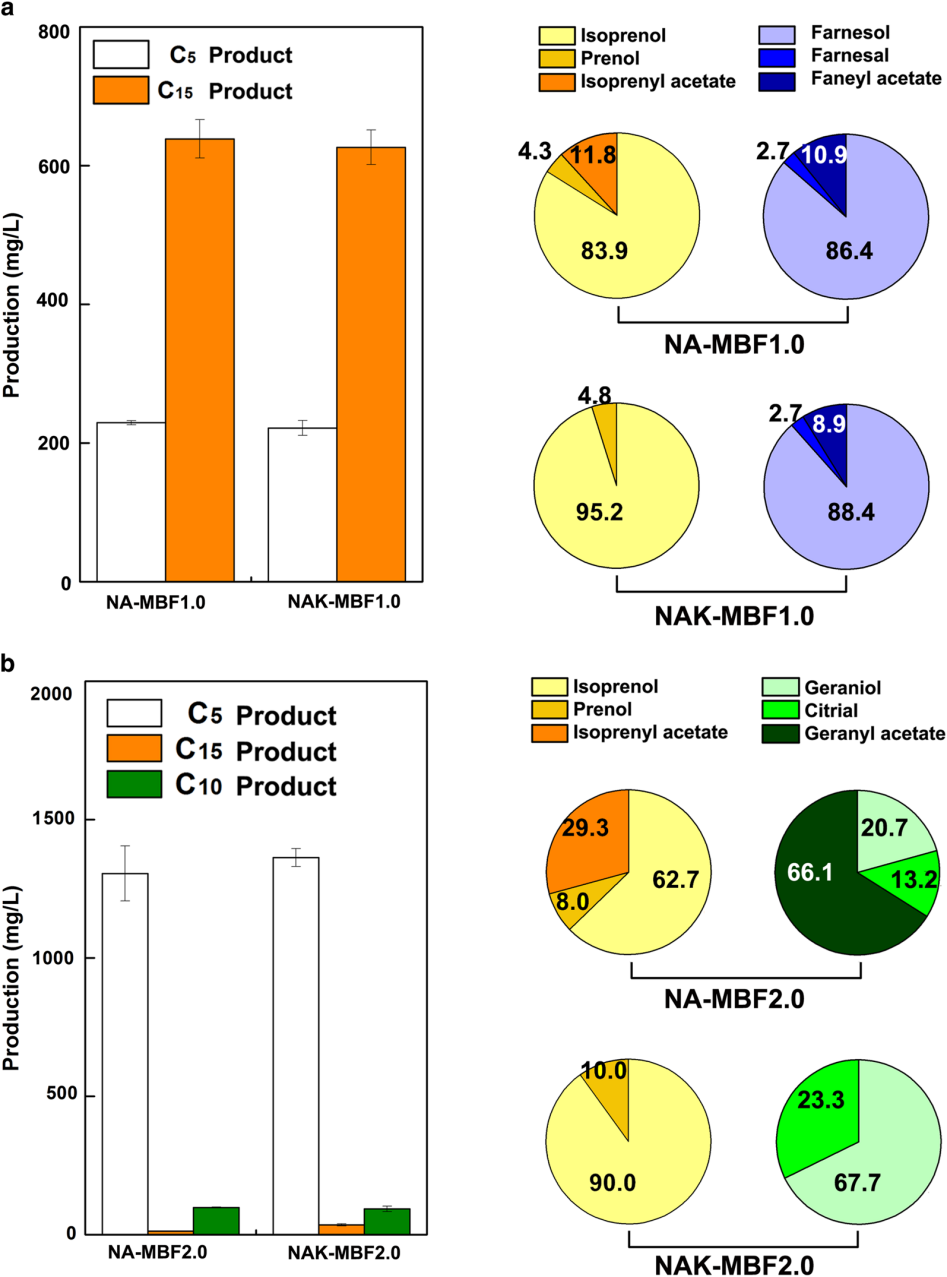
Production of C_5_, C_10_, and C_15_ isoprenoid alcohols and their derivatives in engineered *E. coli*. **a** Production and fractional compositions (%) of IPP/DMAPP (C_5_)- and FPP (C_15_)-derived isoprenoids from strains NA-MBF1.0 and NAK-MBF1.0. Strains NA-MBF1.0 and NAK-MBF1.0 are *E. coli* DH5α strains harboring pSNA and pSNAK, respectively, along with pT-MBF1.0. **b** Production and fractional compositions (%) of IPP/DMAPP (C_5_)-, GPP (C_10_)-, and FPP (C_15_)-derived isoprenoids from strains NA-MBF2.0 and NAK-MBF2.0. Strains NA-MBF2.0 and NAK-MBF2.0 were transformed with pSNA and pSNAK, respectively, along with pT-MBF2.0. Cultures were incubated at 30 °C for 48 h in 2YT medium containing 2.0% (v/v) glycerol and were initially induced with 0.5 mM IPTG. The error bars represent the range from three independent experiments

In order to convert IPP/DMAPP to isopentenol and FPP to farnesol in *E. coli*, it is necessary to overexpress endogenous or exogenous phosphatases for dephosphorylation of these prenyl diphosphates. The endogenous phosphatase NudB effectively hydrolyzes IPP and DMAPP to isopentenol [13, 41–43]. As Nudix hydrolases generally show broad substrate specificities [38], we hypothesized that NudB could also have catalytic activity toward FPP. Thus, *nudB* was cloned with *ispA* encoding FPP synthase into a strong expression vector, pTrc99A, resulting in plasmid pT-MBF1.0 (Fig. 1b). Plasmid pSNA, expressing an exogenous mevalonate (MVA) pathway, was used to effectively supply prenyl diphosphate precursors for synthesis of mixture isoprenoid alcohols (Fig. 1b) [44]. Both of the plasmids were transformed into *E. coli* DH5α, resulting in strain NA-MBF1.0 (Additional file 1: Table S1). After culture of the strain for 48 h, the culture broth was analyzed by gas chromatography (GC), which showed seven peaks that were subsequently identified through gas chromatography–mass spectrometry (GC–MS) (Fig. 2; Additional file 1: Fig. S1). Three of the seven peaks were identified to be isoprenol, prenol (C_5_ alcohols), and farnesol (C_15_ alcohol) as expected (Fig. 2). The other four unexpected peaks corresponded to isoprenyl acetate, farnesyl acetate, *Z*,*E*-farnesal, and *E*,*E*-farnesal (Fig. 2). Thus, the strain NA-MBF1.0 successfully produced 221 mg/L of C_5_ alcohols and their derivatives, and 626 mg/L of a C_15_ alcohol and its derivatives, which was a total of 847 mg/L isoprenoid biofuel mixture (Fig. 3a). In addition to C_5_ products, 65.1% of the total isoprenoid mixture produced in the strain NA-MBF1.0 was farnesol, a fourfold increase over the reference strain (135.5 mg/L) overexpressing IspA only [37]. Previously, the overexpression of NudB was used for the production of C_5_ products, isopentenol, because it was considered to have its activity only to IPP/DMAPP [13, 41–43]. However, our result indicates that NudB is able to dephosphorylate FPP to farnesol (C_15_ products), as well as IPP/DMAPP to isopentenol. NudB was thus presumed to be a potential phosphatase, hydrolyzing a wide range of prenyl diphosphates, and is likely appropriate for production of a mixture of isoprenoid alcohols as biofuel. The new promiscuous activity of NudB toward FPP was verified by in vitro study (Additional file 2: Fig. S6).

### Engineering a synthetic pathway for a mixture of isoprenoid-based C_5_ and C_10_ alcohols

The recombinant *E. coli* strain overexpressing IspA and NudB successfully produced C_5_ and C_15_ isoprenoid alcohols, but not the C_10_ isoprenoid alcohol. This is probably due to absence of a GPP synthase (GPPS) to catalyze the condensation of DMAPP and IPP to GPP, the universal precursor of all monoterpenes (C_10_) (Fig. 1a). Thus, production of C_10_ isoprenoid alcohol in *E. coli* has been limited due to poor supply of GPP. *E. coli* IspA was mutated to release GPP precursor for monoterpene biosynthesis [45]. The mutant IspA (IspA*), with a mutation of serine to phenylalanine at the 80th residue, catalyzes a single condensation of IPP and DMAPP, unlike wild-type IspA that catalyzes two sequential condensations of two IPPs with DMAPP. Overexpression of IspA* and the geraniol synthase of *O. basilicum* in recombinant *E. coli* harboring a heterologous MVA pathway was reported to produce 182 mg/L of geraniol [26]. Thus, IspA* was also used in this study to supply GPP to evaluate the activity of NudB for production of C_10_ isoprenoid alcohol. Plasmid pT-MBF2.0 was constructed by replacement of the *ispA* gene on plasmid pT-MBF1.0 with ispA* (Fig. 1b). *E. coli* strain NA-MBF2.0 harboring pT-MBF2.0 and pSNA produced geraniol and its derivatives (citral and geranyl acetate) as well as isoprenol, isoprenyl acetate, prenol, prenyl acetate, and farnesol (Fig. 2; Additional file 1: Fig. S1). A total concentration of 1418 mg/L of C_5_ and C_10_ products with a small amount (12 mg/L) of C_15_ product was produced from the strain NA-MBF2.0 after 48 h of culture (Fig. 3b; Table 1). The C_5_ family products were a major fraction of the isoprenoid mixture, but the C_10_ and C_15_ family products were minor components (Fig. 3b). The amount of C_10_ family products (99 mg/L) obtained by overexpression of IspA* in strain NA-MBF2.0 was significantly lower than that of the C_15_ family products (626 mg/L) from strain NA-MBF1.0 with overexpression of IspA. This is probably due to the lower catalytic activity of IspA* compared to wild-type IspA. Thus, the lower production of C_10_ family products in strain NA-MBF2.0 could be attributed to a poor supply of GPP. An effective GPPS is necessary for sufficient supply of GPP to increase C_10_ products in the isoprenoid mixture. No study has previously focused on the promiscuous activity of NudB toward GPP. Our findings showed that NudB dephosphorylates GPP to geraniol. The new promiscuous activity of NudB toward GPP was verified by in vitro study (Additional file 2: Fig. S6).

**Table 1:** Production of isoprenoid alcohols from recombinant *E. coli* using NudB, PgpB, and YbjG

Phosphatase	Isopentenol (C_5_, mg/L)	Geraniol (C_10_, mg/L)	Farnesol (C_15_, mg/L)	Total production (mg/L)	References
NudB	55	–	–	55	[13]
NudB	2230	–	–	2230	[27]
NudB	475	–	–	475	[42]
NudB	1130	–	–	1130	[43]
Not specified	–	–	135.5	135.5	[37]
PgpB/YbjG	–	–	526.1	526.1	[49]
NudB	1319	99	12	1430	This study (NA-MBF2.0 strain)
NudB	1105	–	922	2027	This study (NA-MBF1.2 strain)
NudB	412	–	1419	1831	This study (NA-MBF1.2a strain)

The “–” represents “not detectable”

### Identification of enzymes leading to the conversion of alcohols to their derivatives

Isoprenyl acetate and farnesyl acetate are esters of isoprenol and farnesol, respectively. The formation of esters has not been reported in the production of isopentenol and farnesol [13, 37, 41, 46–49]. Chloramphenicol acetyltransferase (CAT) has been reported to possess nonspecific esterification activity toward some isoprenoid alcohols such as perillyl alcohol [50], geraniol [26], and retinol [51]. Thus, the formation of isoprenyl acetate and farnesyl acetate in strain NA-MBF1.0 was suspected due to the chloramphenicol antibiotic marker (Cm^R^) gene (*cat*) in plasmid pSNA. To validate this theory, plasmid pSNAK with a kanamycin antibiotic marker (Km^R^) gene was used instead of pSNA. The strain NAK-MBF1.0 harboring pT-MBF1.0 and pSNAK produced isoprenoid alcohols (isoprenol, prenol, and farnesol), farnesal, and farnesyl acetate, but no formation of isoprenyl acetate was observed (Fig. 3a). However, the production of farnesyl acetate was still observed in strain NAK-MBF1.0 (Fig. 3a). This indicates that CAT contributes to the esterification of isoprenol to its acetyl ester, but is not responsible for the formation of farnesyl acetate. Hence, some unknown endogenous acetyltransferase in *E. coli* seems to be involved in the esterification of farnesol into farnesyl acetate (Fig. 1a). The cell growth of NAK-MBF1.0 was slightly lower than that of NA-MBF1.0 (Additional file 1: Fig. S2).

Farnesal is an oxidized product of farnesol. The oxidation of farnesol to farnesal and even to farnesoic acid by alcohol dehydrogenase has been reported in *Aedes aegypti* [52]. Oxidation of geraniol to geranial and neral by alcohol dehydrogenases has been reported in *E. coli* [26]. The formation of farnesal in strains NA-MBF1.0 and NAK-MBF1.0 was thus suspected to be a result of oxidation of farnesol by some endogenous alcohol dehydrogenases/aldehyde reductases. To address this, YjgB, YahK, YddN, and AdhE from *E. coli* were overexpressed using the vector pTrc99A. YjgB and YahK are NADPH-dependent aldehyde reductases, exhibiting broad substrate specificity toward aldehydes and alcohols [53]. YddN is a NADH-dependent acetaldehyde reductase with a catalytic activity toward medium-chain alcohols [54]. AdhE is an aldehyde-alcohol dehydrogenase, catalyzing the conversion of primary alcohols to aldehydes [55]. Authentic standard farnesol was fed to the culture of *E. coli* strains DH5α-Trc, DH5α-YjgB, DH5α-YahK, DH5α-YddN, and DH5α-AdhE harboring plasmids pTrc99A, pT-YjgB, pT-YahK, pT-YddN, and pT-AdhE, respectively (Additional file 1: Table S2). The strain DH5α-YjgB significantly transformed farnesol to farnesal (Additional file 1: Table S2). To confirm the dehydrogenase activity of YjgB toward farnesol, farnesol was fed to a culture of mutant strain MG*ΔYjgB* with a deletion of *yjgB*; no farnesal peak was observed in GC analysis of the culture (data not shown).

Similarly, formation of isoprenyl, prenyl, and geranyl acetates in strain NA-MBF2.0 was also suspected due to esterification of isoprenol, prenol, and geraniol, respectively, by the CAT of plasmid pSNA. There was no formation of the isoprenoid acetate esters in the strain NAK-MBF2.0 harboring pT-MBF2.0 and pSNAK (Fig. 3b). The citral produced from strains NA-MBF2.0 and NAK-MBF2.0 is the dehydrogenated product of geraniol formed by YjgB [26]. Cell growth of strain NAK-MBF2.0 was slightly reduced in comparison to that of strain NA-MBF2.0 (Additional file 1: Fig. S2).

### Effects of *ispA* and *nudB* operon gene order along with overexpression of *aphA* on total titer of the isoprenoid mixture

**Fig. 4:**
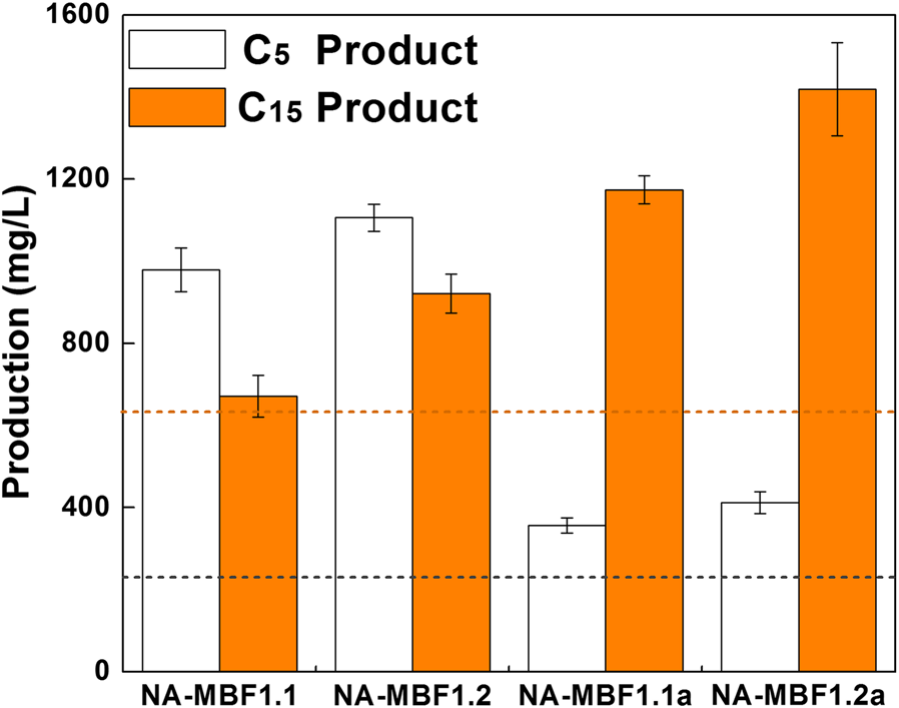
An optimized synthetic pathway for the production of C_5_ and C_15_ isoprenoid alcohols and their derivatives. Production of IPP/DMAPP (C_5_)- and FPP (C_15_)-derived isoprenoids from strains NA-MBF1.1, NA-MBF1.2, NA-MBF1.1a, and NA-MBF1.2a harboring pT-MBF1.1, pT-MBF1.2, pT-MBF1.1a, and pT-MBF1.2a, respectively, in addition to pSNA. Cultures were incubated at 30 °C for 48 h in 2YT medium containing 2.0% (v/v) glycerol and were initially induced with 0.5 mM IPTG. The error bars represent the range from three independent experiments. The two dotted lines represent the production of C_5_ products (black) and C_15_ products (orange) from strain NA-MBF1.0

Since we successfully constructed strains for synthesis of C_5_–C_10_ and C_5_–C_15_ isoprenoid-based biofuel mixtures, we next sought to improve the production of the C_5_–C_15_ isoprenoid-based biofuels mixture. A balanced expression of production pathway genes is critical to achieve high titers of desirable products. There is a fundamental relationship between expression level of genes and their order in an operon. In general, expression of a gene increases as its position in an operon moves closer to a promoter [56]. Positional modulation of the constituent genes in a synthetic operon has significantly enhanced productions of zeaxanthin, taxadiene, and protoilludene in *E. coli* [57–59]. The order of the *ispA* and *nudB* genes in pT-MBF1.0 was reversed to construct pT-MBF1.1 (Fig. 1b). Cell growth of strain NA-MBF1.1 harboring pT-MBF1.1 and pSNA was significantly enhanced in comparison with the growth of strain NA-MBF1.0 (Additional file 1: Fig. S3). The total titer of the isoprenoid mixture from strain NA-MBF1.1 was 1652 mg/L, a twofold increase over the production of strain NA-MBF1.0 (847 mg/L) (Fig. 4). The significant enhancement of the total production was mainly due to the increased production of C_5_ products (prenol, isoprenol, and isoprenyl acetate) from 221 to 979 mg/L, whereas the production of C_15_ products (farnesol, farnesal, and farnesyl acetate) was not significantly different between the strains (Fig. 4). These results show that insufficient expression of NudB from pT-MBF1.0 caused the lower production of C_5_ products in strain NA-MBF1.0 because of the limited conversion of IPP and DMAPP to C_5_ products, which would also cause an intracellular accumulation of IPP and DMAPP. The lower cell growth of strain NA-MBF1.0 was presumed to be due to the accumulation of IPP and DMAPP, which are toxic and can inhibit normal cell growth [60].

NudB was thought to convert IPP and DMAPP directly to isoprenol and prenol, respectively [13]. However, a recent study has shown that NudB hydrolyzes IPP and DMAPP into their monophosphate forms, IP and DMAP, respectively, in *E. coli*, and the subsequent hydrolysis of IP and DMAP to isopentenol is performed by other phosphatases such as AphA, Agp, and YqaB [42]. The *aphA* gene was thus cloned into plasmid pT-MBF1.1 after *ispA*, resulting in pT-MBF1.2 (Fig. 1b). The strain NA-MBF1.2 harboring pT-MBF1.2 and pSNA produced 2027 mg/L of total isoprenoid mixture (1105 mg/L of C_5_ products and 922 mg/L of C_15_ products), which was an increase of 1.2-fold over the production of strain NA-MBF1.1 (Fig. 4). The overexpression of AphA enhanced the production of not only the C_5_ products but also the C_15_ products. This suggests that FPP is sequentially dephosphorylated to farnesol by the cooperation of NudB and AphA, similar to the dephosphorylation of IPP/DMAPP to isopentenol. A significant decrease in farnesol production has been reported in a previous study of an AphA mutant strain [37], indicating the involvement of AphA in farnesol production. The cell growth of strain NA-MBF1.2 was slightly reduced in comparison with that of strain NA-MBF1.1 (Additional file 1: Fig. S3). To date, NudB has been only known to have its activity on IPP/DMAPP and used for production of C_5_ isoprenoid alcohols (Table 1). However, NudB has a promiscuous activity on isoprenyl diphosphates (IPP/DMAPP, GPP, and FPP), and is successfully applied here for production of mixed isoprenoid alcohols (Table 1). Although George et al. reported 2230 mg/L of a single C_5_ alcohol (isoprenol), their production of mixed C_5_ alcohols (isoprenol, prenol, and isopentanol) was only 1150 mg/L [41]. Here, we produced the comparable amount of mixed C_5_ alcohols (1105 mg/L) in addition to C_15_ alcohol (922 mg/L), a total mixed isoprenoid alcohol of 2027 mg/L.

### Augmented expression of the *idi* gene changes the compositional profile of the isoprenoid mixture

**Fig. 5:**
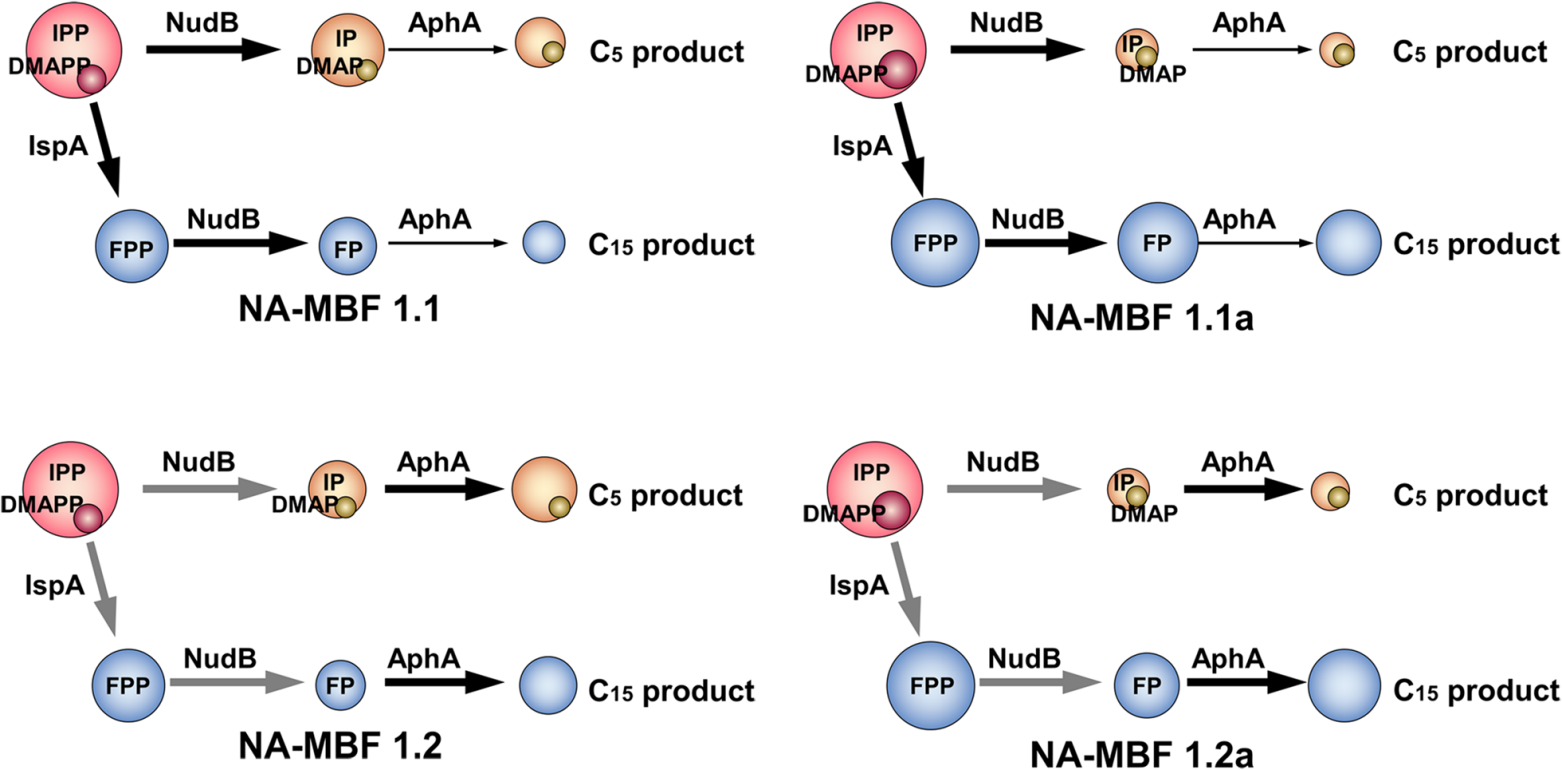
Scheme of isoprenyl diphosphates flux distribution in engineered *E. coli* strains with and without additional overexpression of IDI. In strains NA-MBF1.1 and NA-MBF1.2 with no additional overexpression of IDI (left panel), metabolic flux of IPP is mainly directed toward formation of isoprenol through NudB. In strains NA-MBF1.1a and NA-MBF1.2a with additional overexpression of IDI (right panel), the IPP flux is diverted toward synthesis of FPP by IspA, resulting in production of farnesol as the dominant component. The circle area represents the accumulation levels of the intermediates, and the arrows represent the flux directions and the enzyme expression levels

**Fig. 6:**
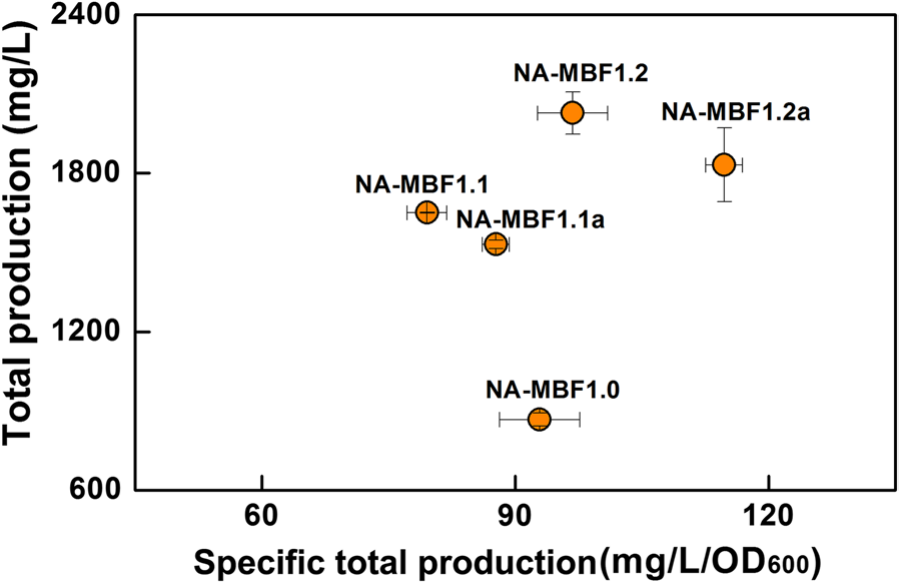
Total production versus specific total production of isoprenoid mixtures from engineered *E. coli* strains. Strains NA-MBF1.1, NA-MBF1.2, NA-MBF1.1a, and NA-MBF1.2a are *E. coli* DH5α strains harboring pT-MBF1.1, pT-MBF1.2, pT-MBF1.1a, and pT-MBF1.2a, respectively, in addition to pSNA. Cultures were incubated at 30 °C for 48 h in 2YT medium containing 2.0% (v/v) glycerol and initially induced with 0.5 mM IPTG. The error bars represent the range from three independent experiments

The prenol fractions of the isoprenoid mixtures obtained from strains NA-MBF1.1 and NA-MBF1.2 were very small compared to those of isoprenol. This is probably due to a lower intracellular level of DMAPP than IPP or a higher specificity of NudB toward IPP than DMAPP. IPP isomerase (IDI) catalyzes the reversible isomerization of IPP to DMAPP and adjusts its ratio to 3:7 [61]. In order to increase the conversion of IPP to DMAPP, the *idi* gene was cloned downstream of the *ispA* gene of pT-MBF1.1 and pT-MBF1.2, resulting in pT-MBF1.1a and pT-MBF1.2a, respectively (Fig. 1b). The effect of the additional overexpression of IDI on the compositional profile of the isoprenoid mixture was investigated in strains NA-MBF1.1a and NA-MBF1.2a harboring pT-MBF1.1a/pSNA and pT-MBF1.2a/pSNA, respectively (Fig. 4; Additional file 1: Fig. S4). The additional overexpression of IDI drastically changed the compositional profile with a significant decrease and increase in C_5_ and C_15_ products, respectively, but a small increase of prenol. IPP-derived products (isoprenol and isoprenyl acetate) of the strains NA-MBF1.1a and NA-MBF1.2a were 303 and 345 mg/L, respectively, which were distinctively lower than 965 and 1086 mg/L of the corresponding strains NA-MBF1.1 and NA-MBF1.2, (Fig. 4, Additional file 1: Fig. S4). The concentrations of DMAPP-derived prenol in strains NA-MBF1.1a and NA-MBF1.2a were 53 and 67 mg/L, respectively, which were increased more than threefold compared to those in strains NA-MBF1.1 and NA-MBF1.2; however, the absolute increases were only 39 and 47 mg/L, respectively (Additional file 1: Fig. S4). However, the concentrations of FPP-derived products (farnesol, farnesal, and farnesyl acetate) of the strains NA-MBF1.1a and NA-MBF1.2a significantly increased to 1174 and 1419 mg/L, respectively, in comparison with 706 and 922 mg/L in strains NA-MBF1.1 and NA-MBF1.2, respectively (Fig. 4; Table 1; Additional file 1: Fig. S4). The increase in FPP-derived products could be ascribed to an increase in the intracellular levels of DMAPP through the expression of IDI. The FPP synthase IspA possesses a higher Km with DMAPP (50 µM) than with IPP (4 µM) [62], and therefore a higher intracellular DMAPP level through the additional overexpression of IDI could be a benefit to divert the flux of isoprenyl diphosphates to FPP. Moreover, the DMAPP would initiate additional FPP synthesis as a reaction-priming molecule (Fig. 5). Although the additional overexpression of IDI increased the prenol fraction in the isoprenoid mixture, there was still a significant difference between the titers of isopentenol (IPP-derived product) and prenol (DMAPP-derived product) (Additional file 1: Fig. S4), which could indicate a preference of NudB for IPP over DMAPP. The cell growths of strains NA-MBF1.1a and NA-MBF1.2a were slightly lower than those of strains NA-MBF1.1 and NA-MBF1.2 (Additional file 1: Fig. S3). The specific total production of the isoprenoid mixtures from strains NA-MBF1.1a and NA-MBF1.2a was enhanced over the corresponding strains NA-MBF1.1 and NA-MBF1.2 (Fig. 6). FPP-derived products were a major fraction of the isoprenoid mixtures obtained from strains NA-MBF1.1a and NA-MBF1.2a. This suggests that NudB is an effective phosphatase even with FPP, and the compositional profile of the isoprenoid mixture can be modulated by changes in metabolic flux toward various isoprenyl diphosphates (Fig. 5). It presents an important issue in field of metabolic engineering. Product profile of an enzyme is often restricted by its surroundings such as an intracellular level of metabolites (intermediates or precursors to the enzymatic reaction), and thus a specificity of enzymes is designated in a given specific condition. This study successfully shows a modulation of product profile by implementations of the promiscuous activity of NudB and a change of intracellular synthesis of the precursor isoprenyl diphosphates (IPP/DMAPP, GPP, and FPP). The maximum titer of farnesol production reported until now is 526 mg/L [49], while we produced 1419 mg/L of farnesol in addition to 412 mg/L of isopentenol. This is the highest titer of farnesol production reported.

## Conclusions

New promiscuous activity of NudB for GPP and FPP was identified for the first time and used for the biosynthesis of isoprenoid mixture biofuels from a cheap carbon source, glycerol, in engineered *E. coli* strains. A mixture of C_5_ and C_15_ isoprenoid alcohols and their derivatives was achieved in recombinant *E. coli* overexpressing NudB and IspA along with an exogenous MVA pathway. The co-overexpression of the acid phosphatase AphA successfully improved production of the C_5_–C_15_ isoprenoid mixture to a maximum titer of 2027 mg/L. The compositional profile of the C_5_–C_15_ isoprenoid mixture can be tailored by overexpression of IDI, resulting in an increase in long carbon chain C_15_ products with higher energy content than the short carbon chain C_5_ products. When IspA was replaced with its mutant IspA* that generates GPP, C_10_ family products were observed in the isoprenoid mixture. All of the isoprenoid mixtures can be good candidates for advanced biofuels and commodity chemicals. This is the first successful report on the production of isoprenoid alcohols and their derivatives in a mixture. This study can serve as the basis for production of other isoprenoid-based mixtures.

## Methods

### Strains, media, and culture conditions

The *E. coli* DH5α strain was used as a host for cloning and production of desired compounds. For plasmids construction, cells were grown overnight at 37 °C in LB (Luria–Bertani) medium (10 g tryptone, 5 g yeast extract, and 10 g sodium chloride per liter), while cultures for seed preparation and isoprenoid production were grown in 2YT medium (16 g tryptone, 10 g yeast extract, and 5 g sodium chloride per liter). Antibiotics were added to the culture medium at a final concentration of 100 mg/L of ampicillin and 50 mg/L of kanamycin or chloramphenicol as required. To make the seed culture, colonies were inoculated in 5 mL of 2YT medium with appropriate antibiotics and grown overnight at 30 °C with shaking at 250 rpm. The production culture was made by inoculating the overnight seed culture into 4 mL of 2YT medium to an optical density at 600 nm (OD_600 nm_) of 0.1 of with 2% (v/v) of glycerol as the main carbon source. The culture was carried out in two-phases by overlaying 1 mL of oleyl alcohol over 4 mL of the culture in order to prevent volatile loss of the products [41, 63]. The two-phase production culture was initially induced with 0.5 mM isopropyl *β*-d-1-thiogalactopyranoside (IPTG) and was incubated for 48 h with 250 rpm shaking in a rotary shaker at 30 °C. Cellular growth of the culture was measured in terms of OD_600 nm_ using a spectrophotometer. Glass tubes (2.5 cm × 15 cm) with plastic caps were used for aerobic culture for all experiments conducted in this study. Genomic DNA of *E. coli* MG1655 was used for PCR amplification of the genes cloned in this study. All strains used in this study are listed in Additional file 1: Table S1.

### Plasmid construction

PCR primers and plasmids used in this study are listed in Additional file 1: Table S1. PCR was performed using *Pfu* DNA polymerase (SolGent, Daejeon, Korea) according to the manufacturer’s instructions for amplification of DNA fragments. The *ispA* [Genbank: NC_000913.3: 440202–441101] fragment was PCR amplified from the genomic DNA of *E. coli* MG1655 with primers IspA-F and IspA-R, digested with restriction enzymes *Sac*I and *Kpn*I, and ligated between the corresponding restriction sites of pTrc99A to create pT-SBL. For the construction of pT-MBF1.0, *nudB* [Genbank: NC_000913.3: 1948180–1948632] was amplified from the genomic DNA with primers NudB-F1 and NudB-R1, digested with BamHI and HindIII, and subsequently inserted between the corresponding restriction sites of pT-SBL. The *ispA* mutant ispA* was PCR amplified with primers IspA*-F and IspA*-R using the plasmid pT-GPSGES [26] as a template. The PCR product was digested with KpnI and BamHI and ligated into the corresponding restriction sites of pT-SBL to create pT-MBF2.0. Another fragment of *nudB* was PCR amplified from pT-MBF1.0 with primers NudB-F2 and NudB-R2, digested with *Nco*I and *Sac*I, and ligated upstream of *ispA* in pT-SBL to create pT-MBF1.1. The *idi* [Genbank: NC_000913.3: 3033065–3033613] fragment was PCR amplified from the genomic DNA with primers Idi-F and Idi-R. The resulting fragment was digested with BamHI and SalI and inserted into the corresponding restriction sites of pT-MBF1.1 to construct pT-MBF1.1a. The DNA fragment of *aphA* [Genbank: NC_000913.3: 4269414–4270127] was PCR amplified from the genomic DNA with primers of AphA-F and AphA-R, digested with *Sal*I and *Hin*dIII, and inserted between the corresponding restriction sites of pT-MBF1.1 and pT-MBF1.1a to create pT-MBF1.2 and pT-MBF1.2a, respectively. The genes overexpressed for transformation of farnesol to farnesal, *yjgB* [Genbank: NC_000913.3: 4495190–4496209], *yahK* [Genbank: NC_000913.3: 342884–343933], and *yddN* [Genbank: NC_000913.3: 1552828–1553838], were previously cloned in the plasmids pT-YjgB, pT-YahK, and pT-YddN, respectively [26]. For construction of pT-AdhE, the DNA fragment of *adhE* [Genbank: NC_000913.3: 1295446–1298121] was PCR amplified from the genomic DNA with primers adhE-F and adhE-R, digested with *Eco*RI and *Bam*HI, and inserted between the corresponding restriction sites of pTrc99A. To convert acetyl-CoA to IPP and DMAPP via the mevalonate (MVA) pathway, plasmid pSNA encoding the MVA pathway was previously constructed by Yoon et al. [44]. The plasmid pSNA was constructed by cloning of the top portion of the MVA pathway (*mvaE* and *mvaS* from *Enterococcus faecalis*) and its bottom portion (*mvaK1*, *mvaK2*, and *mvaD* from *Streptococcus pneumonia*, and *idi* from *E. coli*) into the pSTV28 vector under control of a *lac* promoter. The *cat* gene encoding chloramphenicol acetyltransferase (CAT) in plasmids pSTV28 and pSNA had been replaced with a kanamycin resistance gene, and the resultant plasmids were named pSTV28 K and pSNAK, respectively [26].

### Farnesol feeding of the culture

To investigate the conversion of farnesol to farnesal, 1 mL of decane containing 1 g/L of farnesol was put over 4 mL of 2YT medium, with 2% (v/v) glycerol as a carbon source, which was inoculated with the recombinant strains DH5α-Trc, DH5α-YjgB, DH5α-YahK, DH5α-YddN, or DH5α-AdhE, or the mutant strain MG*ΔYjgB*. The culture of each strain was initially induced with 0.2 mM IPTG and incubated at 30 °C with 250 rpm shaking in a rotary shaker for 48 h. The decane phase was collected at 12-h intervals and subsequently subjected to GC analysis to observe the conversion of farnesol to farnesal.

### Analysis of products by GC–MS and GC-FID

The entire culture broth including oleyl alcohol phase was extracted with ethyl acetate after 48 h of culture [41]. The ethyl acetate fraction was obtained by high-speed centrifugation and subjected to analysis by GC–MS and GC-FID. Formation of isoprenoid mixtures, composed of isoprenol, isoprenyl acetate, prenol, prenyl acetate, geraniol, citral, geranyl acetates, farnesol, farnesal, and farnesyl acetate, was identified by GC–MS (GC–MS-QP2010, SHIMADZU, Japan). All products were quantitatively analyzed using GC (Agilent Technologies 7890A) equipped with a flame ionization detector (FID). Samples were injected into a DB-WAX123-7032 column (30 m in length, 0.320 mm in internal diameter, and 0.25 μm in film thickness), and nitrogen was used as a carrier gas. The GC oven temperature started at 40 °C and was raised with a gradient of 5 °C/min until 80 °C and held for 1 min. The temperature was then raised at a rate of 60 °C/min until 235 °C where it was held for 5 min. The detector temperature was maintained at 260 °C. The standard curves for isoprenol, prenol, isoprenyl acetate, geraniol, citral, geranyl acetates, farnesol, farnesal, and farnesyl acetate were constructed for the quantitative calculation of their production (Additional file 1: Fig. S5).

## Additional files

**Additional file 1: Table S1.** Primers, plasmids and bacterial strains used in this study. **Table S2.** Time course analysis of oxidation of farnesol to farnesal in *E. coli* DH5α-YjgB. **Figure S1.** GC-FID and GC-MS profile of standard isoprenoid-based alcohols and their derivatives. **Figure S2.** Comparison of cell growth of the strains NA-MBF2.0, NAK-MBF2.0, NA-MBF1.0, and NAK-MBF1.0. **Figure S3.** Comparison of cell growth of the strains NA-MBF1.1, NA-MBF1.2, NA-MBF1.1a and NA-MBF1.2a. **Figure S4.** Percent composition of isoprenoid mixtures obtained from the strains NA-MBF1.1, NA-MBF1.2, NA-MBF1.1a and NA-MBF1.2a. **Figure S5.** GC-FID standard curves of isoprenoid alcohols and their derivatives.

**Additional file 2: Table S3.** Primers, plasmids and bacterial strains used in this study. **Figure S6.** GPP and FPP hydrolyzing assay of NudB.

## Abbreviations

IPP: isopentenyl diphosphate
DMAPP: dimethylallyl diphosphate
GPP: geranyl diphosphate
FPP: farnesyl diphosphate
IP: isopentenyl phosphate
DMAP: dimethylallyl phosphate
GP: geranyl phosphate
FP: farnesyl phosphate
MEP: methylerythritol 4-phosphate
MVA: mevalonate
IPTG: isopropyl *β*-d-thiogalactoside
PCR: polymerase chain reaction
GC-FID: gas chromatography-flame ionization detector
GC–MS: gas chromatography–mass spectrometry
