# Supplementary material for: Metabolic engineering of Escherichia coli for production of mixed isoprenoid alcohols and their derivatives
Source: Biotechnol Biofuels. 2018 Jul 24;11:210. doi: 10.1186/s13068-018-1210-0 (PMC6058358; doi:10.1186/s13068-018-1210-0)
Supplement: Supplementary file 1 — Additional file 1: Table S1. Primers, plasmids and bacterial strains used in this study. Table S2. Time course analysis of oxidation of farnesol to farnesal in E. coli DH5α-YjgB. Figure S1. GC-FID and GC-MS profile of standard isoprenoid-based alcohols and their derivatives. Figure S2. Comparison of cell growth of the strains NA-MBF2.0, NAK-MBF2.0, NA-MBF1.0, and NAK-MBF1.0. Figure S3. Comparison of cell growth of the strains NA-MBF1.1, NA-MBF1.2, NA-MBF1.1a and NA-MBF1.2a. Figure S4. Percent composition of isoprenoid mixtures obtained from the strains NA-MBF1.1, NA-MBF1.2, NA-MBF1.1a and NA-MBF1.2a. Figure S5. GC-FID standard curves of isoprenoid alcohols and their derivatives. [file 13068_2018_1210_MOESM1_ESM.docx]

**Additional File 1**

**Metabolic engineering of *Escherichia* *coli* for production of mixed isoprenoid alcohols and their derivatives**

Bakht Zada^a,1^, Chonglong Wang^b,2^, Ji-Bin Park^a^, Seong-Hee Jeong^a^, Ju-Eon Park^a^, Hawaibam Birla Singh^a^, Seon-Won Kim^a*^

^a^Division of Applied Life Science (BK21 Plus Program), PMBBRC, Gyeongsang National University, Jinju 660‑701, Republic of Korea

^b^School of Biology and Basic Medical Sciences, Soochow University, Suzhou, People's Republic of China

^1^Both authors contributed equally to this work.

E-mail: bakhtqau@gmail.com (B.Z.), clwang@suda.edu.cn (C.W.), [jbjnas@hanmail.net](mailto:jbjnas@hanmail.net)

(J.B.P.), [jshe89@nate.com](mailto:jshe89@nate.com) (S.H.J.), [parkwndjs@naver.com](mailto:parkwndjs@naver.com) (J.E.P.),

birla.hawaibam@gmail.com (H.B.S.)

^*^ Corresponding author: Prof. Seon-Won Kim

E-mail: swkim@gsnu.ac.kr (SW Kim), Tel.: +82 55 772 1362, Fax: +82 55 759 9363.

**Table S1.** Primers, plasmids, and bacterial strains used in this study.

**Table S2.** Time course analysis of oxidation of farnesol to farnesal in strain DH5α-YjgB.

**Figure S1.** GC-FID and GC-MS profile of standard isoprenoid-based alcohols and their derivatives.

**Figure S2.** Comparison of the cell growth of strains NA-MBF2.0, NAK-MBF2.0, NA-MBF1.0, and NAK-MBF1.0.

**Figure S3.** Comparison of the cell growth of strains NA-MBF1.1, NA-MBF1.2, NA-MBF1.1a, and NA-MBF1.2a

**Figure S4.** Percent composition of isoprenoid biofuels obtained from strains NA-MBF1.1, NA-MBF1.2, NA-MBF1.1a, and NA-MBF1.2a.

**Figure S5.** GC-FID standard curves of isoprenoid-based alcohols and their derivatives.

| **Names** | **Descriptions** | **References** |
| --- | --- | --- |
| **Primers^a^** |  |  |
| IspA-F | CTAGGAGCTCAGCCCTAAGGAACCAATATGGACTTTCCGCAGCAAC | This study |
| IspA-R | TGAGGTACCTTATTTATTACGCTGGATGATGTAG | This study |
| NudB-F1 | CTGGATCCTTTAAGAAGGAGATATACATATGAAGGATAAAGTGTATAAGCGTCC | This study |
| NudB-R1 | CCCAAGCTTAGGCAGCGTTAATTACAAACTG | This study |
| IspA*-F | GAGGTACCAACAATAATCTCGTATAGTATGGACTTTCCGCAGCAAC | This study |
| IspA*-R | CTGGATCCTTATTTATTACGCTGGATGATGTAG | This study |
| NudB-F2 | CATCCATGGTTTAAGAAGGAGATATACATATGAAGGATAAAGTGTATAAGCGTCC | This study |
| NudB-R2 | CATGGAGCTCTTAGGCAGCGTTAATTACAAACTG | This study |
| AphA-F | TATCGTCGACAAGTCATTCGGGGCCAATTTATGCGCAAGATCACACAGGC | This study |
| AphA-R | CCCAAGCTTAGTATTCTGAATTGACGATCACCTC | This study |
| Idi-F | ACGGATCCTGAGGAGGTAACGTATGCAAACGGAACACGTCATTTTA | This study |
| Idi-R | TATCGTCGACTCTAAGATCTTATTTAAGCTGGGTAAATGCAG | This study |
| AdhE-F | TACGAATTCAGGAGGTAATAATAATGGCTGTTACTAATGTCGC | This study |
| AdhE-R | ACGGATCCTAAAGCGGATTTTTTCGC | This study |
| **Plasmids** |  |  |
| pSTV28 | P*_lac_* expression vector, pACYC184 origin, lacZ, Cm^r^ | Takara Co., Ltd |
| pSNA | pSTV28 containing *mvaE* and *mvaS* of *E. faecalis,* *mvaK1, mvaK2,* and *mvaD* of *S. pneumoniae,* and *idi* of *E. coli* | [[1](#_ENREF_1)] |
| pSTV28K | P*_lac_* expression vector, pACYC184 origin, lacZ, Km^r^ | [[2](#_ENREF_2)] |
| pSNAK | pSTV28K containing *mvaE* and *mvaS* from *E. faecalis*, *mvaK1, mvaK2*, and *mvaD* from *S. pneumoniae*, and idi from *E. coli* | [[2](#_ENREF_2)] |
| pTrc99A | P*_trc_* expression vector, pBR322 origin, lacI^q^, Amp^r^ | [[3](#_ENREF_3)] |
| pT-SBL | pTrc99A vector containing *ispA* from *E. coli* | This study |
| pT-MBF1.0 | pTrc99A vector containing *ispA* and *nudB* from *E. coli* | This study |
| pT-MBF2.0 | pTrc99A vector containing *ispA** and *nudB* from *E. coli* | This study |
| pT-MBF1.1 | pTrc99A vector containing *nudB* and *ispA* from *E. coli* | This study |
| pT-MBF1.2 | pTrc99A vector containing *nudB, ispA* and *aphA* from *E. coli* | This study |
| pT-MBF1.1a | pTrc99A vector containing *nudB, ispA* and *idi* from *E. coli* | This study |
| pT-MBF1.2a | pTrc99A vector containing *nudB, ispA*, *idi* and *aphA* from *E. coli* | This study |
| pT-YahK | pTrc99A vector containing *yahK* from *E. coli* | [[2](#_ENREF_2)] |
| pT-YddN | pTrc99A vector containing *yddN* from *E. coli* | [[2](#_ENREF_2)] |
| pT-AdhE | pTrc99A vector containing *adhE* from *E. coli* | This study |
| pT-YjgB | pTrc99A vector containing *yjgB* from *E. coli* | [[2](#_ENREF_2)] |
| **Strains** |  | [[2](#_ENREF_2)] |
| MG1655 | *E. coli* K-12; F^-^ lambda^-^, ilvG^-^, rfb-50, rph-1 | ATCC 700926^b^ |
| DH5α | *E. coli* K-12; F^–^, Φ80lacZΔM15, Δ(lacZYA-argF)U169, deoR, recA1, endA1, hsdR17(rK^–^, mK^+^) phoA, supE44, λ^–^, thi-1 | ATCC 98040 |
| NA-MBF1.0 | *E. coli* DH5α harboring pT-MBF1.0 and pSNA | This study |
| NAK-MBF1.0 | *E. coli* DH5α harboring pT-MBF2.0 and pSNA | This study |
| NA-MBF2.0 | *E. coli* DH5α harboring pT-MBF1.0 and pSNAK | This study |
| NAK-MBF2.0 | *E. coli* DH5α harboring pT-MBF2.0 and pSNAK | This study |
| NA-MBF1.1 | *E. coli* DH5α harboring pT-MBF1.1 and pSNA | This study |
| NA-MBF1.2 | *E. coli* DH5α harboring pT-MBF1.2 and pSNA | This study |
| NA-MBF1.1a | *E. coli* DH5α harboring pT-MBF1.1a and pSNA | This study |
| NA-MBF1.2a | *E. coli* DH5α harboring pT-MBF1.2a and pSNA | This study |
| DH5α-Trc | *E. coli* DH5α harboring pTrc99A | This study |
| DH5α -YjgB | *E. coli* DH5α harboring pT-YjgB | This study |
| DH5α -Yahk | *E. coli* DH5α harboring pT-Yahk | This study |
| DH5α -Yddn | *E. coli* DH5α harboring pT-YddN | This study |
| DH5α -Adhe | *E. coli* DH5α harboring pT-AdhE | This study |
| MG*ΔYjgB* | MG1655 *ΔYjgB* | [[2](#_ENREF_2)] |

**Table S1.**

**Primers, plasmids, and bacterial strains used in this study.**

^a^Restriction enzyme sites are underlined.

^b^ATCC, American type culture collection.

**Table S2.**

**Time course analysis of oxidation of farnesol to farnesal in strain DH5α-YjgB.**

| Time (h) | Farnesol (mg/L) | Farnesal (mg/L) | Total (mg/L) |
| --- | --- | --- | --- |
| 12 | 979 ± 5.6 | 10 ± 1.5 | 989 ± 6.6 |
| 24 | 956 ± 6.3 | 28 ± 2.6 | 984 ± 8.9 |
| 36 | 896 ± 9.1 | 89 ± 7.2 | 985 ± 16.3 |
| 48 | 873 ± 7.1 | 116 ± 4.2 | 989 ± 11.3 |

*Farnesol was fed in a two-phase culture of strain DH5α-YjgB at a concentration of 1 g/L. The culture was initially induced with 0.2 mM IPTG and incubated at 30°C with 250 rpm shaking in a rotary shaker for 48 h. Decane phase was collected at 12 h intervals and subsequently subjected to GC for analysis of compositional variations between farnesol and farnesal. Results are presented as the means of the triplicate experiments (M ± SD).

**
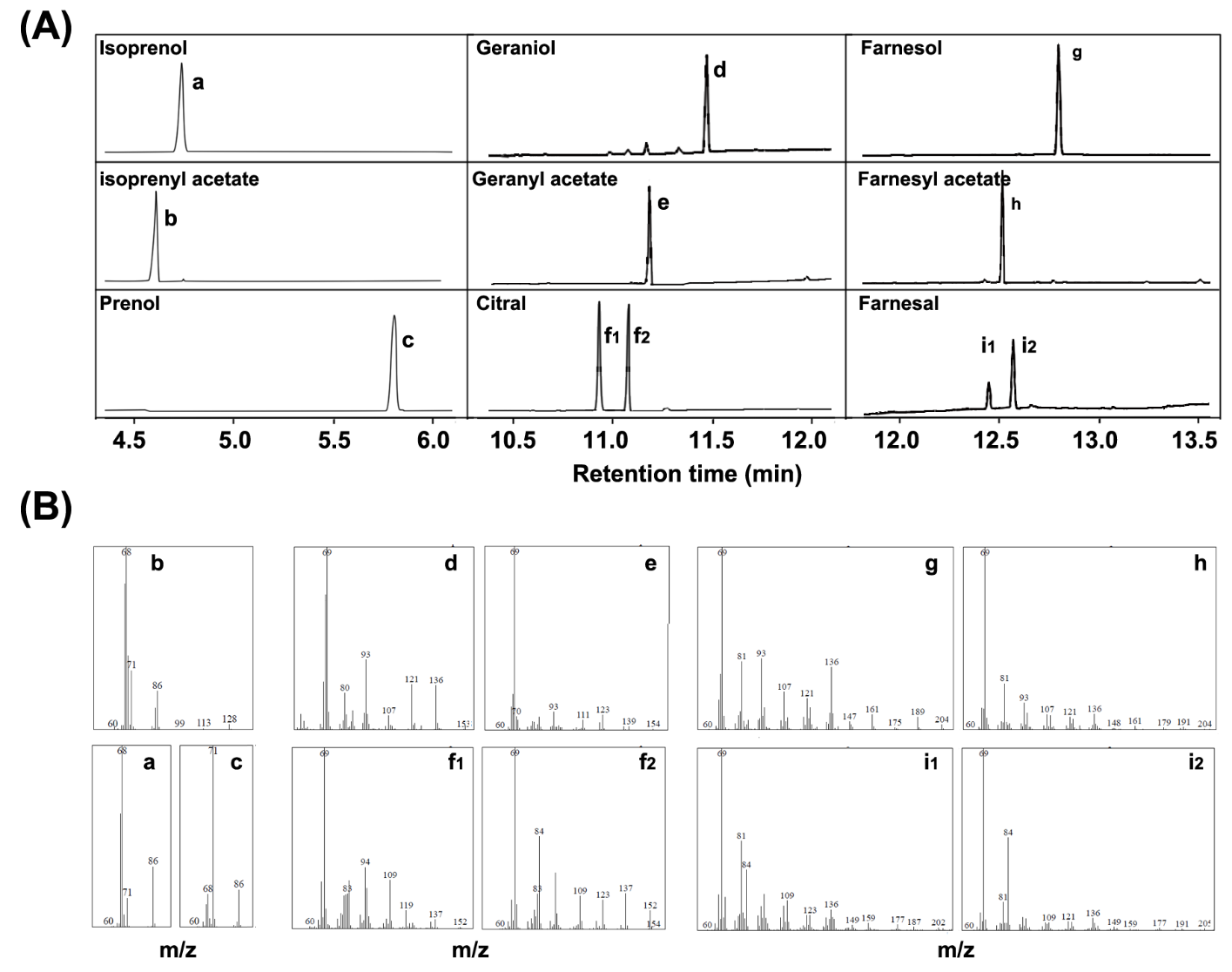
Figure S1.**

**GC-FID and GC-MS profile of standard isoprenoid alcohols and their derivatives.** GC chromatograms **(A)** and mass spectra **(B)** of standard isoprenoid compounds. Standard citral exists as isomers, *E*-citral (f_1_) and *Z*-citral (f_2_). Standard farnesal also exists as isomers of *Z*,*E*-farnesal (i_1_) and *E*,*E*-farnesal (i_2_).


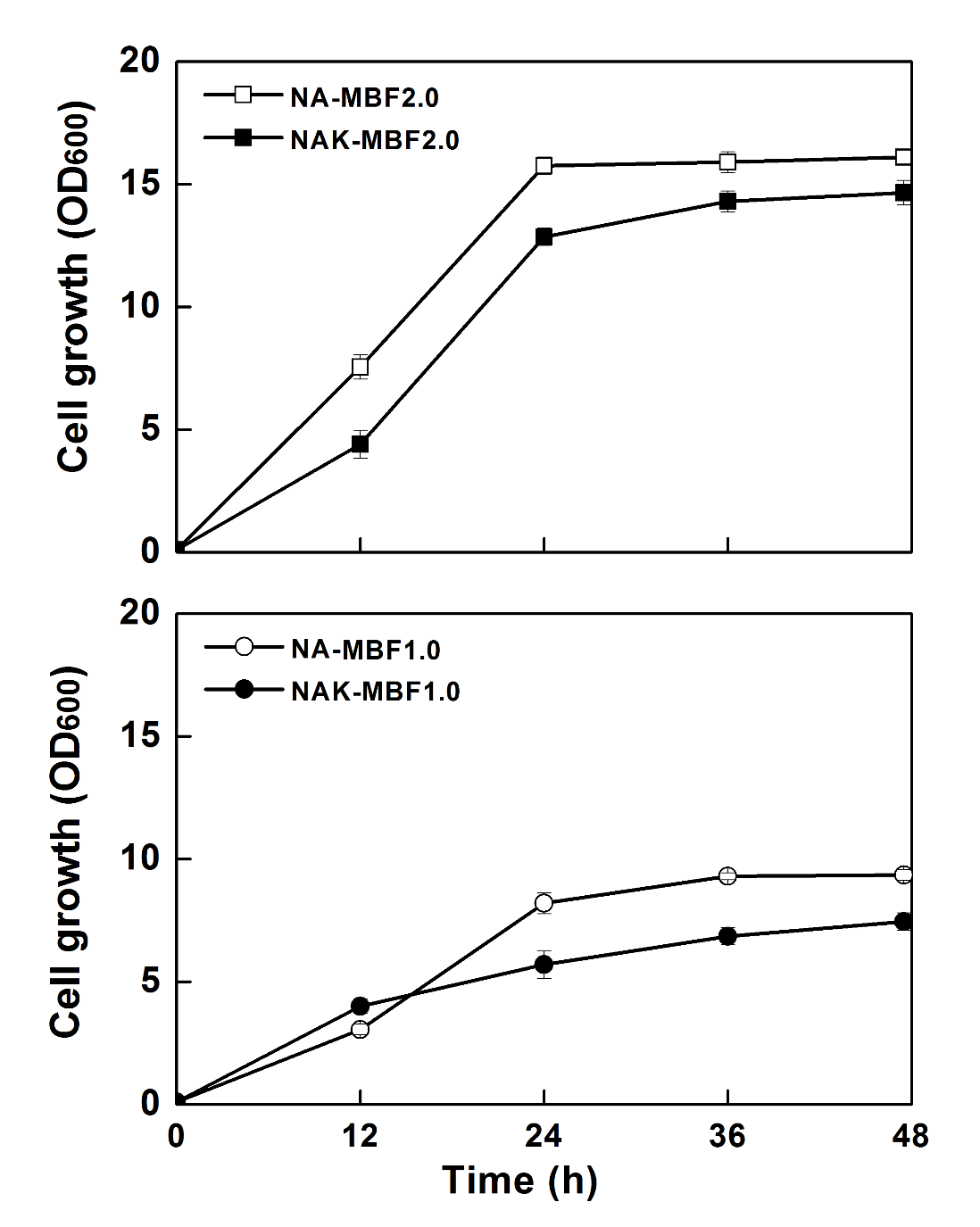


**Figure S2.**

**Comparison of cell growth of strains NA-MBF2.0 and NAK-MBF2.0 (upper panel) and the strains NA-MBF1.0 and NAK-MBF1.0 (lower panel).** Strains NA-MBF2.0 and NAK-MBF2.0 are *E. coli* DH5α harboring pSNA and pSNAK, respectively, in addition to pT-MBF2.0; strains NA-MBF1.0 and NAK-MBF1.0 harbor pT-MBF1.0 instead of pT-MBF1.0. Strains were cultured at 30°C for 48 h in 2YT medium containing 2.0% (v/v) glycerol and were initially induced with 0.5 mM IPTG. Error bars represent the range from three independent experiments.

**

Figure S3.**

**Comparison of cell growth of strains NA-MBF1.1, NA-MBF1.2, NA-MBF1.1a, and NA-MBF1.2a harboring NA-MBF1.1, NA-MBF1.2, NA-MBF1.1a, and NA-MBF1.2a, respectively, in addition to pSNA.** The dotted line represents the cell growth of strain NA-MBF1.0. The strains were cultured at 30°C for 48 h in 2YT medium containing 2.0% (v/v) glycerol and were initially induced with 0.5 mM IPTG. The error bars represent the range from three independent experiments.

**Figure S4.**

**
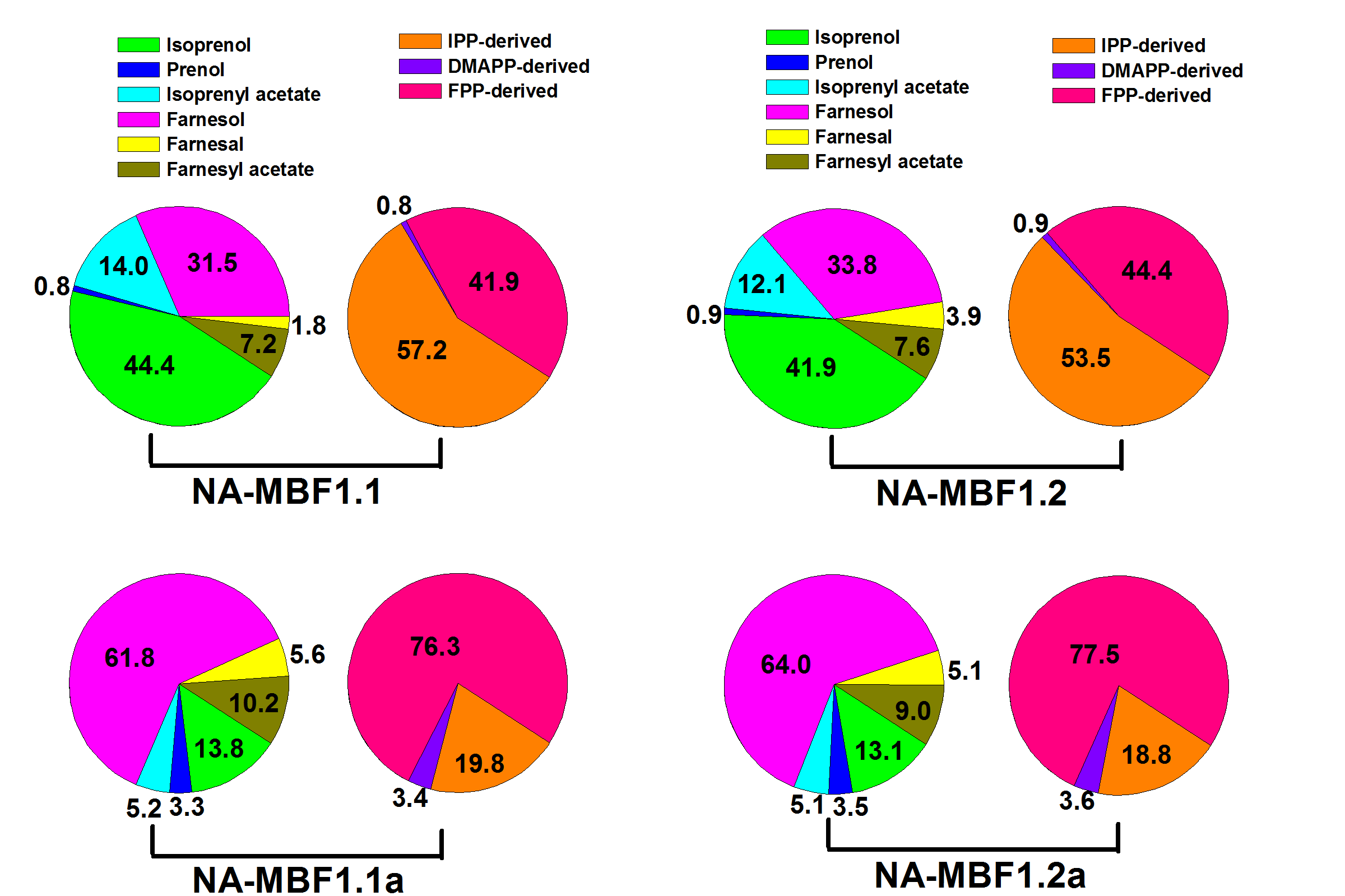
**Percent composition of isoprenoids mixtures obtained from strains NA-MBF1.1, NA-MBF1.2, NA-MBF1.1a, and NA-MBF1.2a. The strains NA-MBF1.1 and NA-MBF1.2 have no additional overexpression of IDI, while the strains NA-MBF1.1a and NA-MBF1.2a have additional overexpression of IDI. Strains were cultured at 30°C for 48 h in 2YT medium containing 2.0% (v/v) glycerol and were initially induced with 0.5 mM IPTG.

**
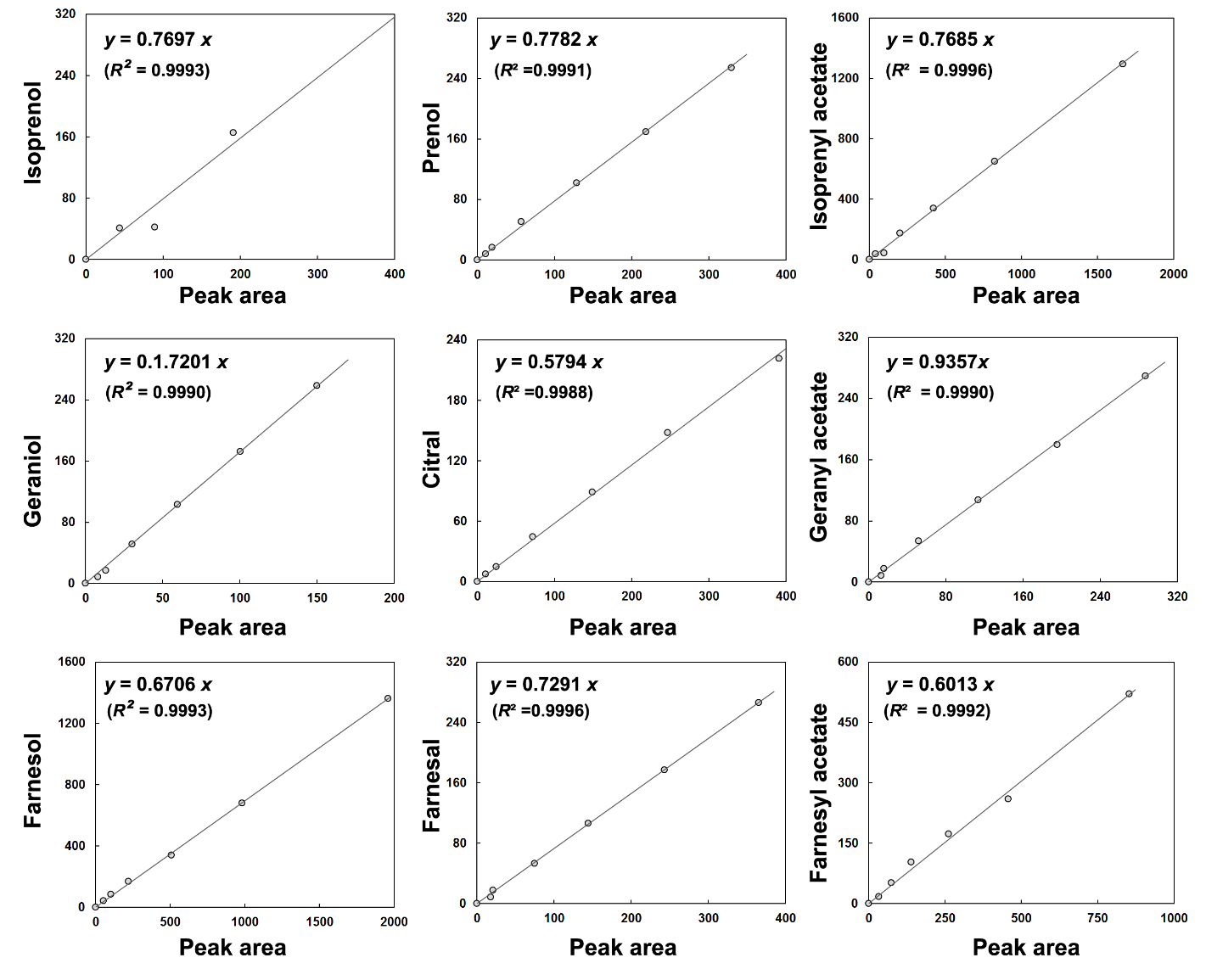
**

**Figure S5.**

**GC-FID standard curves of isoprenoid alcohols and their derivatives.** All standard curves were generated using the means of three replicated experiments. The x-axis represents the GC peak area of the isoprenoids, while the y-axis represents the concentration of the corresponding standard compound in ethyl acetate.

**References**

1. Yoon SH, Lee SH, Das A, Ryu HK, Jang HJ, Kim JY, Oh DK, Keasling JD, Kim SW: **Combinatorial expression of bacterial whole mevalonate pathway for the production of beta-carotene in *E. coli***. *Journal of biotechnology* 2009, **140**(3-4):218-226.

2. Zhou J, Wang C, Yoon SH, Jang HJ, Choi ES, Kim SW: **Engineering *Escherichia coli* for selective geraniol production with minimized endogenous dehydrogenation**. *Journal of biotechnology* 2014, **169**:42-50.

3. Amann E, Ochs B, Abel KJ: **Tightly regulated tac promoter vectors useful for the expression of unfused and fused proteins in *Escherichia coli***. *Gene* 1988, **69**(2):301-315.
