# Supplementary material for: Metabolic engineering of Escherichia coli for production of mixed isoprenoid alcohols and their derivatives
Source: Biotechnol Biofuels. 2018 Jul 24;11:210. doi: 10.1186/s13068-018-1210-0 (PMC6058358; doi:10.1186/s13068-018-1210-0)
Supplement: Supplementary file 2 — Additional file 2: Table S3. Primers, plasmids and bacterial strains used in this study. Figure S6. GPP and FPP hydrolyzing assay of NudB. [file 13068_2018_1210_MOESM2_ESM.docx]

**Additional File 2**

**Metabolic engineering of *Escherichia* *coli* for production of mixed isoprenoid alcohols and their derivatives**

Bakht Zada^a,1^, Chonglong Wang^b,1^, Ji-Bin Park^a^, Seong-Hee Jeong^a^, Ju-Eon Park^a^, Hawaibam Birla Singh^a^, Seon-Won Kim^a*^

^a^Division of Applied Life Science (BK21 Plus Program), PMBBRC, Gyeongsang National University, Jinju 660‑701, Republic of Korea

^b^School of Biology and Basic Medical Sciences, Soochow University, Suzhou, People's Republic of China

^1^Both authors contributed equally to this work.

E-mail: bakhtqau@gmail.com (B.Z.), clwang@suda.edu.cn (C.W.), [jbjnas@hanmail.net](mailto:jbjnas@hanmail.net)

(J.B.P.), [jshe89@nate.com](mailto:jshe89@nate.com) (S.H.J.), [parkwndjs@naver.com](mailto:parkwndjs@naver.com) (J.E.P.),

birla.hawaibam@gmail.com (H.B.S.)

^*^ Corresponding author: Prof. Seon-Won Kim

E-mail: swkim@gsnu.ac.kr (SW Kim), Tel.: +82 55 772 1362, Fax: +82 55 759 9363.

**Table S3.** Primers, plasmids, and bacterial strains used in this study

**Figure S6.** GPP and FPP hydrolyzing assay of NudB

**Preparation of FPP substrate**

In order to extract an intracellular FPP, we transformed *E. coli* DH5α with pSSN12Didi and pT-IspA plasmids, which overexpress mevalonate bottom pathway and farnesyl pyrophosphate synthase (IspA), respectively, resulting in strain DH5-FPP (Table 1). A single colony from DH5-FPP strain was inoculated in 5 mL LB medium with appropriate antibiotics and grown overnight at 37°C with shaking at 250 rpm. The FPP production culture was made by inoculating the overnight seed culture into 2YT medium to an OD of 0.1 with 5 mM mevalonate, and 2% (v/v) of glycerol as a main carbon source. The culture was initially induced with 0.2 mM IPTG and incubated for 36 h with 250 rpm shaking in a rotary shaker at 30°C. After 36 h of incubation, cells were harvested by centrifugation. The supernatant was discarded and the cells pellets were suspended in lysis buffer consisted of 2% glycerol, 0.1M NaCl, 0.025M Tris-HCl (pH 8.0), and 0.2% triton X-100, and subsequently lysed by sonication to extract FPP. The cell lysates were incubated at 65°C for 30 min in order to inactivate the enzymes.

**Quantification of FPP extracted from culture of DH5-FPP strain**

It has been reported that calf intestinal alkaline phosphatase (CIP) dephosphorylates FPP to farnesol [1]. In order to quantify FPP in the lysate of DH5-FPP culture, we incubated 1mL lysate with CIP for 2 h at 37°C. After 2 h of incubation, the reaction medium was extracted with decane, and subsequently analyzed by GC-FID. In addition, 1mL of lysate from DH5-FPP strain without CIP digestion was taken as control. By the incubation of the cell lysate with CIP, 73.8 µM (16.4 mg/L) of farnesol was obtained. On the other hand, no farnesol was detected in the control sample (without CIP digestion). The amount of farnesol obtained from the CIP reaction could directly reflect the amount of FPP present in the cell lysate. In order to get a lucrative amount of FPP for *in vitro* reaction of NudB, the cell lysate solution was concentrated by 10-fold with use of vacuum concentrator (Concentrator plus Eppendorf with integrated diaphragm vacuum pump). The concentrated lysate was quantified to 725.4 µM (161.3 mg/L) of farnesol after the treatment of CIP.

**Preparation of cells lysates for *in vitro* reaction of NudB**

To study the dephosphorylation activity of NudB toward GPP and FPP, NudB was overexpressed under a strong trc promoter in pT-NudB plasmid. For construction of pT-NudB, the DNA fragment of *nudB* was PCR amplified from the genomic DNA of *E. coli* MG1655 with primers NudB-F and NudB-R (Table 1), digested with NcoI and SacI, and inserted between the corresponding restriction sites of pTrc99A. The pT-NudB plasmid was transformed into *E. coli* DH5α, resulting in strain DH5-NudB (Table 1). Stain DH5-Trc, *E. coli* DH5α harboring empty pTrc99A vector, was taken as a control (Table 1). A single colony from each individual strain was inoculated in 5 mL LB medium with appropriate antibiotics and grown overnight at 37°C with shaking at 250 rpm. The overnight culture was further inoculated into fresh medium to an OD of 0.1 with 0.5 mM IPTG and were grown for 12 h at 30°C, shaking at 250 rpm. After 12 h, culture from each strain with the same density was pelleted by centrifugation at 4°C, and was suspended in 1 mL lysis buffer containing 50 mM Tris-HCl (pH 8.0), 1 mM DTT, 1% glycerol, and 10 mM NaCl. After the cells were lysed by sonication, the crude cell lysates were used for *in vitro* reactions of NudB.

**GPP and FPP hydrolyzing assay of NudB**

To verify the new promiscuous activity of NudB, the cell lysates from each individual strain, DH5-Trc and DH5-NudB was incubated with either a commercial GPP obtained from Sigma (CAS Number 763-10-0) or the crude FPP achieved from culture of DH5-FPP strain, where the cell lysates and the substrate (GPP or FPP) solutions were mixed in 1:1 volume ratio. After incubating for 1 h at 37°C, the assay reactions were extracted with decane, and subsequently analyzed by GC-FID to quantify the amount of geraniol and farnesol generated from GPP and FPP, respectively. The cell lysate from the strain DH5-NudB overexpressing NudB produced a significant amounts of geraniol (554.5 µM) and farnesol (305.2 µM) from GPP (600.0 µM) and FPP (362.7 µM), respectively (Fig. S1). On the other hand, the cell lysate from the control strain, DH5-Trc produced a small amount of geraniol and farnesol from GPP and FPP, respectively. The results demonstrate that NudB has a strong catalytic activity toward GPP and FPP.

**Table S3. Primers, plasmids, and bacterial strains used in this study.**

| **Names** | **Descriptions** | **References** |
| --- | --- | --- |
| **Primers** |  |  |
| NudB-F | CATCCATGGTTTAAGAAGGAGATATACATATGAAGGATAAAGTGTATAAGCGTCC | This study |
| NudB-R | CATGGAGCTCTTAGGCAGCGTTAATTACAAACTG | This study |
| **Plasmids** |  |  |
| pSTV28 | P*_lac_* expression vector, pACYC184 origin, lacZ, Cm^r^ | Takara Co., Ltd |
| pSSN12Didi | pSTV28 containing *mvaK1, mvaK2*, and *mvaD* from *S. pneumoniae*, and idi from *E. coli* | [2] |
| pTrc99A | P*_trc_* expression vector, pBR322 origin, lacI^q^, Amp^r^ | Amersham Bioscience |
| pT-NudB | pTrc99A vector containing *nudB* from *E. coli* | This study |
| **Strains** |  |  |
| DH5α | *E. coli* K-12; F^–^, Φ80lacZΔM15, Δ(lacZYA-argF)U169, deoR, recA1, endA1, hsdR17(rK^–^, mK^+^) phoA, supE44, λ^–^, thi-1 | ATCC 98040 |
| DH5-FPP | *E. coli* DH5α harboring pSSN12Didi and pT-IspA | This study |
| DH5-Trc | *E. coli* DH5α harboring pTrc99A | This study |
| DH5-NudB | *E. coli* DH5α harboring pT-NudB | This study |

**Figure S6. GPP and FPP hydrolyzing assay of NudB.** **(A)** Production of geraniol from 600.0 µM of GPP by cell lysates of DH5-Trc and DH5-NudB strains for 1 h incubation **(B)** Production of farnesol from 362.7 µM of FPP by cell lysates of DH5-Trc and DH5-NudB strains for 1 h incubation. Strains DH5-Trc and DH5-NudB are *E. coli* DH5α harboring pTrc99A and pT-NudB, respectively.


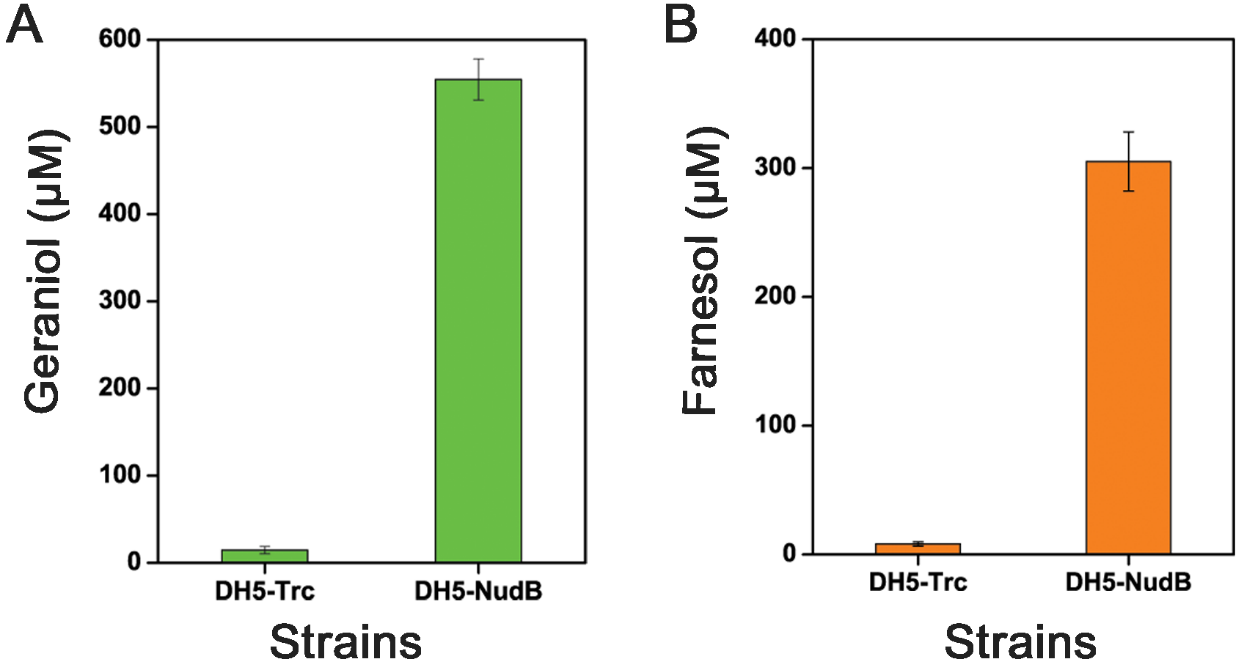


**References**

1. Mackie H, Overton KH: **Hydrolysis and isomerization of trans,trans-farnesyl diphosphate by Andrographis tissue-culture enzymes**. *Eur J Biochem* 1977, **77**(1):101-106.

2. Yoon SH, Lee YM, Kim JE, Lee SH, Lee JH, Kim JY, Jung KH, Shin YC, Keasling JD, Kim SW: **Enhanced lycopene production in Escherichia coli engineered to synthesize isopentenyl diphosphate and dimethylallyl diphosphate from mevalonate**. *Biotechnol Bioeng* 2006, **94**(6):1025-1032.
